# *Staphylococcus aureus* Nuc2 Is a Functional, Surface-Attached Extracellular Nuclease

**DOI:** 10.1371/journal.pone.0095574

**Published:** 2014-04-21

**Authors:** Megan R. Kiedrowski, Heidi A. Crosby, Frank J. Hernandez, Cheryl L. Malone, James O. McNamara, Alexander R. Horswill

**Affiliations:** 1 Department of Microbiology, Roy J. and Lucille A. Carver College of Medicine, University of Iowa, Iowa City, Iowa, United States of America; 2 Department of Internal Medicine, Roy J. and Lucille A. Carver College of Medicine, University of Iowa, Iowa City, Iowa, United States of America; Universitätsklinikum Hamburg-Eppendorf, Germany

## Abstract

*Staphylococcus aureus* is a prominent bacterial pathogen that causes a diverse range of acute and chronic infections. Recently, it has been demonstrated that the secreted nuclease (Nuc) enzyme is a virulence factor in multiple models of infection, and *in vivo* expression of *nuc* has facilitated the development of an infection imaging approach based on Nuc-activatable probes. Interestingly, *S. aureus* strains encode a second nuclease (Nuc2) that has received limited attention. With the growing interest in bacterial nucleases, we sought to characterize Nuc2 in more detail through localization, expression, and biochemical studies. Fluorescence microscopy and alkaline phosphatase localization approaches using Nuc2-GFP and Nuc2-PhoA fusions, respectively, demonstrated that Nuc2 is membrane bound with the C-terminus facing the extracellular environment, indicating it is a signal-anchored Type II membrane protein. Nuc2 enzyme activity was detectable on the *S. aureus* cell surface using a fluorescence resonance energy transfer (FRET) assay, and in time courses, both *nuc2* transcription and enzyme activity peaked in early logarithmic growth and declined in stationary phase. Using a mouse model of *S. aureus* pyomyositis, Nuc2 activity was detected with activatable probes *in vivo* in *nuc* mutant strains, demonstrating that Nuc2 is produced during infections. To assess Nuc2 biochemical properties, the protein was purified and found to cleave both single- and double-stranded DNA, and it exhibited thermostability and calcium dependence, paralleling the properties of Nuc. Purified Nuc2 prevented biofilm formation *in vitro* and modestly decreased biomass in dispersal experiments. Altogether, our findings confirm that *S. aureus* encodes a second, surface-attached and functional DNase that is expressed during infections and displays similar biochemical properties to the secreted Nuc enzyme.

## Introduction

*Staphylococcus aureus* is a notorious pathogen known to cause numerous acute and chronic diseases [1]–[3]. The diversity of the tissues affected and infection potency is due in part to the many secreted virulence factors produced by *S. aureus*. These secreted factors include toxins, superantigens, and a suite of exoenzymes: proteases, lipases, hyaluronidase, phospholipase, and nuclease. There is speculation that the enhanced production of these secreted factors may correlate with the pathogenesis of *S. aureus* strains [4]–[6], particularly in community-associated methicillin-resistant *S. aureus* (CA-MRSA) strains [4], .

In recent years, there has been renewed interest in the *S. aureus* nuclease (Nuc) enzyme. This enzyme was originally identified in 1956 by Cunningham [9] and was named “micrococcal nuclease” (also called “thermonuclease”), and in subsequent years, Nuc became a target of intense biochemical study at the enzymatic and structural level [10]–[14]. More recently, progress has been made towards identifying the biological role of Nuc, and it was demonstrated that this enzyme is a virulence factor in pneumonia and invasive murine models of disease [15], [16]. Like DNases produced by other Gram-positive species, Nuc is required for the evasion of neutrophil extracellular traps (NETs) [15]. Nuc also has a role in the inhibition of biofilm formation through the cleavage of extracellular DNA (eDNA) and prevention of biofilm initiation [17], [18]. Most recently, the broad substrate specificity of Nuc has been exploited to track *S. aureus* infections *in vivo* using activatable probes [19].

The chromosome of *S. aureus* encodes a nuclease-like homolog, called Nuc2, which has received some recent attention [20]–[23]. Nuc2 is functional when expressed in heterologous hosts [23], and initial tests indicate that it is active in biochemical assays [20]. In contrast to Nuc, there does not appear to be a significant role for Nuc2 in biofilm development [22]. This information led us to perform a more thorough investigation of the Nuc2 enzyme. We found that Nuc2 is an active, surface-localized enzyme in *S. aureus*, and we confirmed that the biochemical properties of Nuc2 mirror those of Nuc. We investigated expression both *in vitro* and *in vivo*, and we tested the impact of purified Nuc2 on *S. aureus* biofilms. We anticipate that the results presented herein will guide future studies to determine the physiological and pathogenic role of Nuc2 in *S. aureus*.

## Materials and Methods

### Ethics statement

The animal studies were reviewed and protocol approved by the University of Iowa Institutional Animal Care and Use Committee. The University of Iowa is AAALAC accredited, and the centralized facilities meet and adhere to the standards in the “Guide and Care of Laboratory Animals.”

### Strains and growth conditions

Bacterial strains and plasmids used are described in Table 1. *E. coli* cultures were grown in Luria-Bertani (LB) broth or on LB agar plates. *S. aureus* strains were grown in tryptic soy broth (TSB) or on tryptic soy agar (TSA) plates unless otherwise indicated. Difco methyl green DNase test agar used to examine extracellular nuclease production was prepared according to manufacturer's instructions (BD, Sparks, MD). Plasmids in *E. coli* were maintained using 100 µg/mL of ampicillin (Amp). Plasmids in *S. aureus* were maintained using 10 µg/mL of chloramphenicol (Cm); 10 µg/mL of erythromycin (Erm); or 10 µg/mL of tetracycline (Tet) when necessary. Liquid cultures were incubated at 37°C with shaking at 200 RPM unless otherwise noted.

**Table 1:** Bacterial strains and plasmids.

Strain or Plasmid	Description	Source or reference
Strains		
*E. coli* strains		
BW25141	Cloning strain	[55]
ER2566	Protein expression strain	New England Biolabs
KS272	*phoA*- host strain	[56]
*S. aureus* strains		
RN4220	Restriction deficient cloning host	[57]
AH1263	USA300 CA-MRSA Erm^S^ (LAC*)	[58]
AH1680	AH1263 *nuc*::LtrB	[18]
AH2496	COL *nuc*::LtrB	[18]
AH2497	MW2 *nuc*::LtrB	[18]
AH2495	Newman *nuc*::LtrB	[18]
AH2494	RN4220 *nuc*::LtrB	[18]
AH2498	SF8300 *nuc*::LtrB	[18]
AH2499	TCH1516 *nuc*::LtrB	[18]
UAMS-1	Osteomyelitis isolate	[59]
AH893	UAMS-1 *nuc*	[60]
AH1200	UAMS-1 *nuc2*::erm	[60]
AH1201	UAMS-1 *nuc nuc2*::erm	[60]
AH3051	AH1263 *nuc*::LtrB *nuc2*::erm	This work
AH3057	AH1263 *nuc2*::erm	This work
Plasmids		
pCM1	*S. aureus* expression plasmid	[58]
pCM1-*nuc2*-sGFP	Nuc2-sGFP translational fusion	This work
pDSW438	pBAD33::'phoA	[30]
pDSW439	pBAD33::ftsI-‘phoA	[30]
pET22b	*E. coli* T7 expression vector	Novagen
pET22b-‘*nuc2*	Truncated Nuc2 expression vector	This work
pCM11	*E. coli*-*S. aureus* shuttle vector	[61]
pMK8	pCM11 + NucSS/Nuc2 catalytic-His	This work
pMK9	pCM11 + NucB/Nuc2 catalytic-His	This work
pMK10	pCM11 + Nuc2TM/NucA catalytic-His	This work
pMK11	pCM11 + Nuc2TM/NucB catalytic-His	This work
pMK12	pCM11 + Nuc-His	This work
pMK13	pCM11 + Nuc2-His	This work

### Recombinant DNA and genetics

Plasmid DNA was prepared from *E. coli* and electroporated into *S. aureus* RN4220 as previously described [24]. DNA was moved from RN4220 into other *S. aureus* strains through transduction with bacteriophage 80α or 11 [25]. All restriction enzymes and enzymes for DNA modification were purchased from New England Biolabs (Beverly, MA) and used according to manufacturer's instructions. Oligonucleotides were synthesized by Integrated DNA Technologies (Coralville, IA) and are listed in Supporting Table S1. Non-radioactive sequencing was performed at the University of Iowa DNA Sequencing Facility.

### Bioinformatics and protein threading

Sequence comparison of Nuc and Nuc2 was performed using a ClustalW alignment of amino acid sequences in SeqBuilder (DNASTAR, Inc). The TOPCONS web server was used to generate Nuc2 topology predictions [26]. Threading of the Nuc2 catalytic domain amino acid sequence onto the Nuc crystal structure was carried out using the Phyre2 web server [27], and the threaded structure was displayed using PyMOL Molecular Graphics System, Version 1.5.0.4 (Schrödinger, LLC).

### Fluorescence microscopy of Nuc2-GFP translational fusion

#### Construction of Nuc2-GFP fusion

A Nuc2-sGFP translational fusion was constructed by 2-step PCR. The *nuc2* gene with 300 base pairs of the upstream native promoter region was amplified by PCR from AH1263 genomic DNA with the primers CLM342 and CLM348 (Supplemental Table 1). The gene that encodes super-folder GFP (*gfp*) was amplified by PCR with the primers CLM344 and CLM332. PCR was performed with a mixture of 1 µL of each previous PCR product, and the overlap product was amplified with the outside primers CLM342 and CLM332. The resulting P*_nuc2_-nuc2*-GFP PCR product was purified, digested with HindIII and EcoRI, and ligated into the vector pCM1, cut with the same enzymes. The resulting plasmid pCM1-*nuc2*-*sGFP* was electroporated into strain RN4220 and moved by phage transduction into *S. aureus* LAC.

#### Fluorescence microscopy

Fluorescence micrograph images of *S. aureus* expressing Nuc2-GFP fusions and cytoplasmic GFP were recorded on an Olympus BX60 microscope equipped with a 100x UPlanApo objective (numerical aperture, 1.35) and captured using a Spot 2 cooled, charge-coupled device camera (Diagnostic Instruments, Sterling Heights, MI) and Image-Pro software version 4.1 (Media Cybernetics, Silver Spring, Md.). Filter settings were used as previously described [28], and samples were prepared for microscopy as previously described [29]. Briefly, microscope slides were prepared with gel pads consisting of 40 µL of 1% agarose in water, spotted onto slides when melted and allowed to cool and set before use. 10 µL of bacterial culture was spotted onto an agarose pad and covered with a glass cover slip prior to imaging.

### Alkaline phosphatase-Nuc2 fusion and assay

#### Construction of alkaline phosphatase fusions

The *nuc2* gene was amplified by PCR from AH1263 genomic DNA using the oligonucleotides MRK7 and MRK8. The *spsB* gene (*S. aureus* positive control) was also amplified by PCR using the oligonucleotides MRK9 and MRK10. The PCR products were purified, digested by SacI and XbaI and ligated to pDSW438 [30] cut by the same enzymes, thereby placing *nuc2*/*phoA* and *spsB*/*phoA* under the control of an arabinose-inducible promoter.

#### PhoA plate assay

The *phoA* minus *E. coli* strain KS272 was used as the host strain to determine the orientation of Nuc2 in the cell membrane. Overnight cultures grown in LB supplemented with chloramphenicol were streaked onto LB Cm agar plates containing arabinose and 5-Bromo-4-chloro-3-indolyl phosphate (BCIP) (Sigma-Aldrich). Plates were incubated overnight at 37°C and observed for the appearance of a blue (positive) or white (negative) phenotype.

### Nuc2 purification

#### Cloning of nuc2 into pET22b

The portion of *nuc2* encoding amino acids N26-K177 was amplified from AH1263 genomic DNA with the primers MRK44 and MRK45. The resulting PCR product was purified, digested with BamHI and XhoI and ligated into the expression vector pET22b (Novagen), cut with the same enzymes. The resulting pET22b-*nuc2* construct was sequenced and moved into *E. coli* strain ER2566 (New England Biolabs), creating strain AH2591.

#### Purification of Nuc2

The *E. coli* strain AH2591 was used for the overexpression and purification of Nuc2. A culture of AH2591 was grown overnight in LB supplemented with Amp, and a 1∶500 dilution was inoculated into 2 L of LB with Amp and grown shaking at 30°C. After reaching an OD_600_ of 0.7, expression was induced with 1 mM IPTG. Following 4 hr of induction, cells were harvested by centrifugation at 12,000×g for 15 min and suspended in 60 mL of equilibration buffer containing 50 mM sodium phosphate, pH 8.0, 0.3 M sodium chloride and 10 mM imidazole. Cells were lysed with 6 mL 10x BugBuster (Novagen) with agitation. Insoluble debris was removed by centrifugation at 20,000×g for 30 min, and the lysate was loaded onto a 5 mL column containing HIS-Select HF Nickel Affinity Gel (Sigma) equilibrated with equilibration buffer, pH 8.0. The affinity gel was washed with 2 volumes deionized water and 3 volumes of equilibration buffer. Lysate was loaded onto the column and washed with equilibration buffer, and Nuc2 was eluted with a buffer consisting of 50 mM sodium phosphate, pH 8.0, 0.3 M sodium chloride and 250 mM imidazole. Fractions containing protein were monitored by Bradford assays and SDS-PAGE. Following dialysis in 1X PBS, Nuc2 protein was diluted with 80% glycerol to a final concentration of 10% glycerol and stored at −80°C.

### FRET activity assays

Nuc enzyme was purchased from Worthington Biochemical (Lakewood, NJ). Fluorescence resonance energy transfer (FRET) assays were performed to measure activity of purified Nuc2 protein as previously described, with Nuc2 protein preparations substituting for spent culture media [18]. To measure Nuc2 activity on the surface of *S. aureus* cells, the liquid FRET assay was modified as follows. Overnight cultures of *S. aureus* strains were grown in TSB shaking at 37°C, sub-cultured into TSB to an initial OD_600_ of 0.05 and grown for an additional 22 hr with shaking at 37°C. The OD_600_ of each subculture was measured, and the cultures were diluted to obtain 1 mL of bacterial suspension equivalent to an OD_600_ of 2.0. Bacteria were harvested by centrifugation at 4,000 RPM in a Microfuge18 Centrifuge (Beckman Coulter) for 3 min, and cells were washed twice in 1X sterile TBS and re-suspended in 100 µL of 1X TBS. Samples were transferred to wells in a black, clear-bottom microtiter plate (Corning), and measurements were obtained by mixing 25 µL of whole washed cells in TBS with 25 µL of 2 µM FRET substrate. Measurements were made over a period of 30 min, with a reading every two min followed by shaking to prevent settling of cells. Fluorescence was measured, and an estimate of the relative Units of Nuc activity per mL was calculated as previously described [18]. Briefly, a standard curve of Nuc activity was generated using purified Nuc enzyme (Worthington). Cell surface-associated nuclease activity was measured using washed cells diluted to an OD_600_ of 2 and the resulting activities were converted to mU/ml of Nuc activity. Values represent means and standard deviations of three or four biological replicates.

### Agarose gel DNA degradation assays

Reaction mixtures (10 µL) were prepared consisting of either strain AH1263 genomic DNA, *E. coli* strain AH14 genomic DNA, double-stranded plasmid DNA (vector pCM11 purified from *E. coli*), single-stranded oligonucleotide DNA fragments (sequence: 5′-GGGGACAAGTTTGTACAAAAAAGCAGGCTTTAAGGGTAGCTTACCAGCACCAC-3′), or salmon-sperm DNA (New England Biolabs). Purified Nuc2 (45 µg) was added to the reaction and incubated for 5 min. As a control, purified Nuc (Worthington) (20 U/mL) was used and incubated with the same DNA substrates in the presence of 10 mM CaCl_2_. Following incubation for 10 min at room temperature, reactions were stopped by the addition of 0.1 volume of 10% SDS. Samples were loaded and electrophoresed on 1% TAE agarose gels, and gels were stained with ethidium bromide and imaged using a GelDock 2000 (BioRad). To test the ability of chelators to block Nuc2 activity, 10 mM EGTA or EDTA was substituted for water in the reaction mixture, and Nuc2 protein was added. Double-stranded plasmid DNA was added to the reaction (total reaction volume 10 µL), and the reactions were incubated for 10 min at room temperature and separated on agarose gels as described above. To determine the specific divalent cation required by Nuc2 for activity, the Nuc2 preparation was stripped of cations by incubation with Chelex100 chelating resin (BioRad). Non-hydrated resin was added to sterile water at a concentration of 0.5 mg/mL, mixed with the Nuc2 sample in a microfuge tube and rocked gently at room temperature for 1 hr. Resin was pelleted by centrifugation at 13,000×g, and the sample was filtered through a SpinX microfuge filter column. DNA reaction mixtures were prepared with *E. coli* genomic DNA and supplemented with either 10 mM CaCl_2_, 10 mM MgCl_2_ or 10 mM ZnCl_2_ buffers. The stripped Nuc2 sample was added and enzymatic activity was tested on agarose gels as described above. To determine thermostability of Nuc2, samples of Nuc, Nuc2, and Bovine DNaseI (Fermentas) were incubated at 75°C for 1 hr.

### Construction of the nuc2 transcriptional reporter

To place the sGFP reporter under *nuc2* promoter control, the promoter region was amplified by PCR from AH1263 genomic DNA using the oligonucleotides MRK39 and MRK38 (Supplemental Table 1). The PCR product was purified, digested by HindIII and KpnI and ligated to pCM11 cut by the same enzymes. The plasmid was confirmed by DNA sequencing, and the final plasmid was designated pCM21.

### Construction and testing of Nuc/Nuc2 chimeras

#### Cloning of Nuc/Nuc2 chimeras

Fragments of *nuc* and *nuc2* were amplified by PCR from AH1263 genomic DNA with the primers described in Supplemental Table 1. To build *nuc*/*nuc2* chimeric genes, DNA fragments encoding N- or C-terminal domains of Nuc and Nuc2 were amplified, and DNA sequence overlapping the desired N- or C-terminal partner fragment was added to the reverse primer (for N-terminal fragments) or forward primer (for C-terminal fragments). Following amplification, fragments were purified with a Gel/PCR DNA Fragments Extraction Kit (IBI Scientific, Peosta, IA). Overlap PCR was performed by adding 1 µL of purified DNA encoding the N-terminal portion of the protein and 1 µL of DNA encoding the C-terminal portion of the protein to the reaction mixture, and the fused gene product was amplified with outside primers. The resulting chimeric genes were purified, digested with KpnI and EcoRI and ligated into pCM11, also cut by the same enzymes.

#### Preparation and testing of supernatant and membrane fractions

Nuc and Nuc2 chimera constructs were transformed into *S. aureus* strain AH1680. Each transformed strain was grown 15 hr in 5 mL of TSB supplemented with Erm while shaking at 37°C in. 500 µL of each culture was filtered using Spin-X Centrifuge Tube Filters (Costar, Corning, NY), and the culture supernatants were saved. Membrane fractions were prepared as previously described [31]. Bacterial culture supernatants and membrane fractions were tested for nuclease activity using a FRET assay as previously described [18].

#### Immunoblots for His-tagged Nuc and Nuc2 chimera proteins

Immunoblots were performed on bacterial culture supernatants and membrane fractions containing chimera constructs with C-terminal His_6_ tags. Filtered culture supernatants were mixed 1∶5 with SDS-PAGE loading buffer and boiled for 10 min, while membrane fraction preparations were mixed 1∶1 with SDS-PAGE loading buffer and were not boiled. 12 µL of each sample was electrophoresed on 12% SDS-PAGE. Proteins were transferred to Immobilon-P PVDF membranes (Millipore) with a Protean III system (BioRad). PVDF membranes were blocked overnight as previously described [18], and proteins were detected by addition of α-Penta-His-HRP direct conjugate (Quiagen) at 1∶4000 in TBST with 5% milk at room temperature for 2 hr. Following washing with TBST, blots were developed using the SuperSignal West Pico Chemiluminescent Substrate (Pierce), followed by exposure to X-ray film.

### Whole cell and culture supernatant nuclease assays with TT probe

Fluorescence plate reader assays were carried out as described [32]. Briefly, for each reaction, 1 µL of a stock solution of TT probe (50 µM concentration [19]) was combined with 9 µL of each sample (buffer, buffer plus recombinant nuclease, TSB culture media, culture supernatant, and washed bacteria concentrated 10-fold (i.e., 1 mL of bacteria culture re-suspended in 0.1 mL of PBS)) and incubated at 37°C for 60 min. 290 µL of PBS supplemented with 10 mM EDTA and 10 mM EGTA was added to each and 95 µL of each diluted reaction was loaded per well into a 96-well plate (96F non-treated black microwell plate (NUNC)). Fluorescence levels were measured with an Analyst HT fluorescence plate reader (LJL Biosystems). Background fluorescence levels of TT probe incubated in buffer or broth, and autofluorescence levels of the supernatant and re-suspended cells were determined and subtracted from the probe-activation reaction values. Dulbecco's phosphate-buffered saline (DPBS) containing physiological levels of calcium and magnesium was obtained from Invitrogen (Carlsbad, CA).

### Mouse pyomyositis model

*S. aureus* cultures were prepared for injection into mice as follows. 5 mL of TSB cultures of UAMS-1 WT, UAMS-1 *nuc* and UAMS-1 *nuc nuc2* were grown overnight at 37°C with shaking at 200 rpm, and each strain was sub-cultured 1∶100 into 5 mL of fresh media and grown for another 12 hr at 37°C with shaking. For injection into mice, bacteria were washed once with PBS and re-suspended in PBS for a cell density of ∼2×10^8^ CFU/mL. To determine bacterial concentrations, serial dilutions of the bacteria were plated on TSA and incubated at 37°C. To infect the mice, 50 µl of 2×10^8^ CFU/mL (1×10^7^ CFU total) was injected intramuscularly (thigh muscle) in female C57BL6 mice (6–8 week old) under isoflurane anesthesia. Mice were shaved before the injections. A total of 9 mice were used; images are representative of 3 independent experiments using 1 mouse per condition. The mice were imaged and sacrificed 48 hours following bacterial injections.

### Imaging evaluation of Nuclease-activated TT probe

Epifluorescence imaging was performed with a Xenogen IVIS 200 imaging system (Caliper). Mice were anesthetized with 2% isoflurane gas anesthesia and placed on an imaging platform inside the optical system for dorsal imaging. Epifluorescence images were acquired with 1 second exposure times and Cy5.5 excitation and emission filters. Fluorescence images were acquired prior to and following (see figure for time-points) tail-vein injections of TT probe. For systemic administration of the probe, 3 nmol of TT probe diluted in PBS were injected via tail vein in a total volume of 120 µL [19]. IVIS 4.2 software was used for image acquisition and analysis and to prepare pseudo-colored overlays of fluorescence and grayscale images. Imaging of mouse tissues following sacrifice (at 45 min time point) and dissection was carried out as described above, but with an adjusted field of view. The gross lesions were photographed with a digital camera after removal of the skin.

### Microtiter plate biofilm assays

Microtiter biofilm assays were performed according to methods previously described [18]. 6 microtiter plate wells were measured per strain per trial, and experiments were repeated in triplicate. For prevention of biofilm and dispersal assays, *S. aureus* strain SH1001 (Δ*agr*::TetM) was used to assess the ability of Nuc2 to disrupt a robust biofilm. For assays examining prevention of biofilm formation, purified Nuc2 was added to microtiter wells at the specified concentrations prior to inoculation of the plate with bacterial culture. Biofilms were then allowed to grow for approximately 15 hr in the presence of Nuc2. For dispersal assays, a separate microtiter plate was inoculated for each time point assessed. Specified concentrations of purified Nuc2 were added to microtiter wells after biofilms had been allowed to establish for 15 hr, and biofilm plates were incubated in the presence of Nuc2 for an additional 30 min or 4 hr.

## Results

### Comparison of Nuc and Nuc2

**Figure 1:**
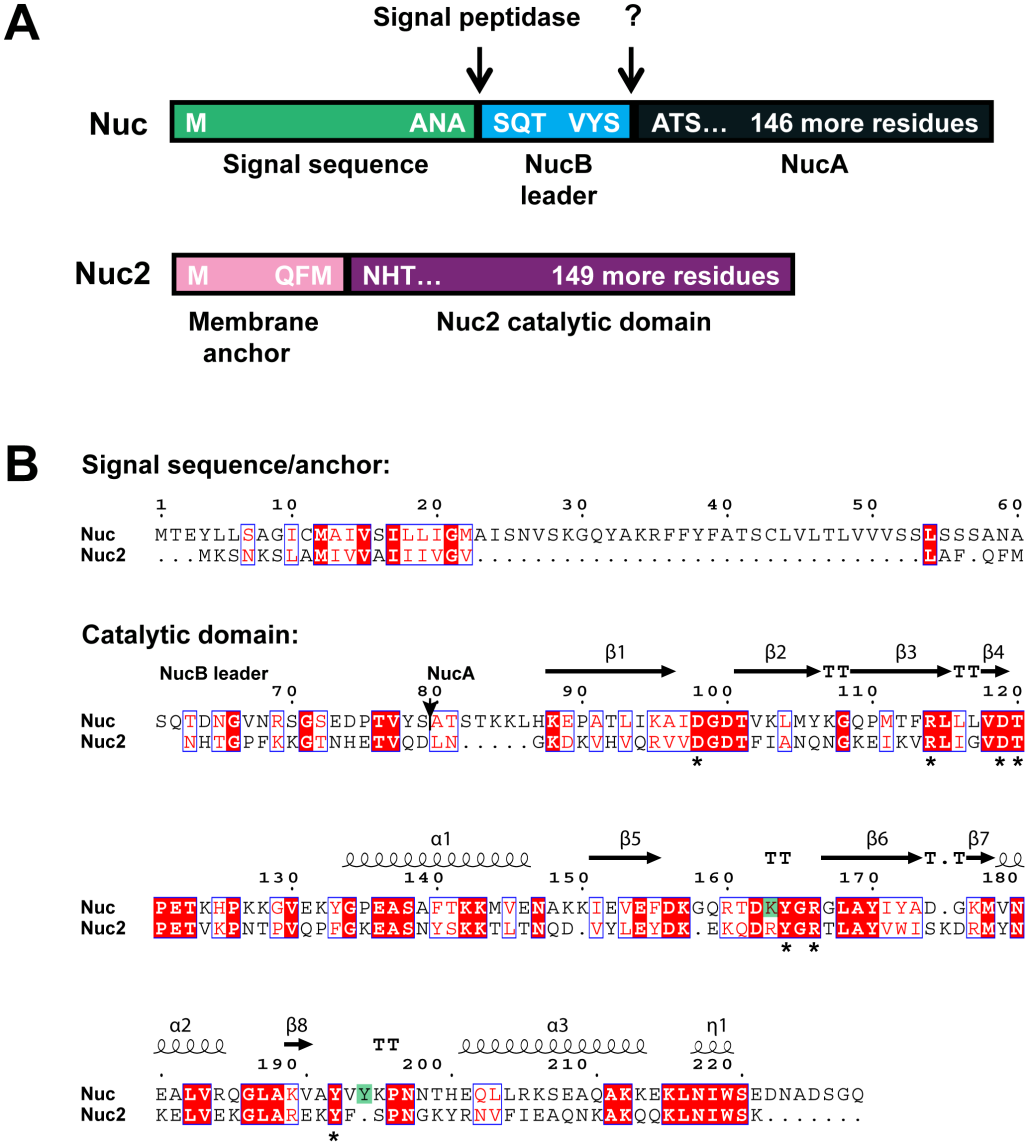
Sequence and structural comparison of the Nuc and Nuc2 proteins. **A**. Schematic of Nuc and Nuc2 protein domains structure, with processing sites shown. The Nuc protein is pictured in shades of teal and blue, showing the N-terminal signal sequence (light teal) cleaved by signal peptidase upon secretion, and the NucB leader (light blue) processed by an unknown protease to release the shorter active form NucA (dark teal). The Nuc2 protein is pictured in shades of purple and depicts the N-terminal membrane anchor (light purple) and the C-terminus containing the SNase active domain (purple). **B**. Amino acid alignment of Nuc (SA0776) and Nuc2 (SA1222) generated with Clustal Omega [52]. The Nuc signal sequence and Nuc2 membrane anchor regions are separated from the catalytic domain as indicated, and the location of NucA start is shown with an arrow at position 80. Structural information (β-sheets and α-helices) was added with ESPript 3.0 [53] based on PDB entry 1EY0 for Nuc [54]. Identical residues are shaded red with white letters, and residues with similar properties are in red font surrounded by blue boxes. Asterisks underneath the alignment indicates residues conserved in Nuc and Nuc2, and the green shading indicates residues present only in Nuc.

In the community associated methicillin-resistant *S. aureus* (CA-MRSA) USA300 genome [33], the *nuc* and *nuc2* genes correspond to ORFs SAUSA300_0776 and SAUSA300_1222, respectively. The genes are separated from one another on the *S. aureus* chromosome by approximately 470 kb. Nuc is a secreted enzyme that possesses an unusually long 60-residue Sec signal sequence. The secreted form of Nuc, known as NucB, is processed by most *S. aureus* strains to a shorter form called NucA (Fig. 1A). Both forms are enzymatically active forms of the protein [18], [34]. The topology of the Nuc2 protein was predicted with computational algorithms (TOPCONS [26]), which suggest it has an N-terminal membrane anchor with the C-terminal enzyme domain facing out of the cell. The protein is thus predicted to be a signal-anchored Type II membrane protein [35]. The Nuc2 enzyme domain shares 42% identity with the Nuc staphylococcal nuclease (SNase) domain (Fig. 1B), and site-directed mutagenesis studies have demonstrated that 9 amino acid residues within the Nuc SNase domain are necessary for maximum hydrolytic activity. Seven of these are conserved in the Nuc2 SNase domain (Fig. 1B). By threading the Nuc2 amino acid sequence onto a crystal structure of Nuc (PDB-1SNO) using Phyre2 [27], there is a high confidence level in the structural similarity of the enzymatic domains of the two proteins (data not shown), supporting observations made recently by Hu et al. [20]. A depiction of the structural relatedness of Nuc and Nuc2 is shown with the sequence alignment in Figure 1B.

### Nuc2 is localized to the cell membrane and faces out of the cell

**Figure 2:**
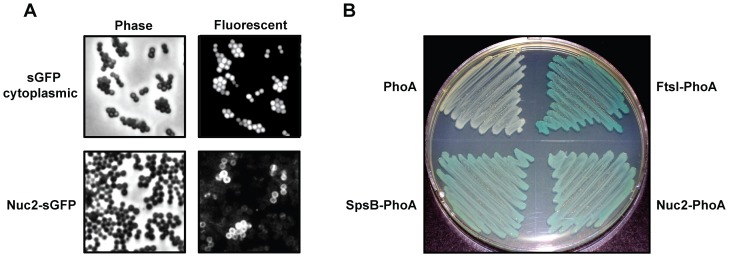
Localization and orientation of Nuc2 in the cell membrane. **A**. Phase and fluorescent microscopy of *S. aureus* expressing cytoplasmic sGFP or Nuc2-sGFP translational fusion. **B**. Alkaline phosphatase fusions grown in a PhoA dye indicator plate assay, where growth appearing white indicates PhoA is facing the cytoplasm and blue indicates PhoA is exposed to the periplasm. Shown clockwise from the upper left corner are: the *E. coli phoA* mutant host, an *E. coli* positive control protein fusion (FtsI-PhoA), the *S. aureus* Nuc2-PhoA protein fusion, and a *S. aureus* positive control protein fusion (SpsB-PhoA).

Computational predictions suggest that Nuc2 is membrane-bound (see Figure 1), and we set out to test this experimentally. Superfolder GFP (sGFP) was appended to the C-terminus of full-length Nuc2 to create a fusion protein. This construct was expressed in *S. aureus* and localization was assessed with fluorescence microscopy. A *S. aureus* strain expressing cytoplasmic sGFP served as a control. The Nuc2-GFP fusion localized to the perimeter of individual *S. aureus* cells, while the cytoplasm is devoid of fluorescence (Fig. 2A). Conversely, cells expressing untagged sGFP show fluorescence limited to the cytoplasm. These observations indicate that Nuc2 is membrane localized.

A previous report demonstrated that expression of *S. aureus nuc2* in *E. coli* resulted in activity on DNase agar plates [36], suggesting that Nuc2 was functional outside of the cell. Having established that Nuc2 is membrane localized, we tested whether the active domain faced the extracellular environment by fusing alkaline phosphatase (PhoA) to the C-terminus of Nuc2 [37]. The Nuc2-PhoA fusion was expressed in an *E. coli phoA* mutant background, and PhoA activity was detected using LB agar plates containing the membrane-impermeable substrate 5-Bromo-4-chloro-3-indolyl phosphate (BCIP). An *E. coli* FtsI-PhoA fusion, in which PhoA is oriented towards the outside of the cell, served as a positive control, and as expected, this strain exhibited a bright blue color on BCIP agar (Fig. 2B). For an *S. aureus* positive control, PhoA was fused to the C-terminus of the type I signal peptidase SpsB [31], and this construct also exhibited a blue color, albeit lighter than the *E. coli* FtsI control. Finally, *E. coli* expressing Nuc2-PhoA appeared light blue, with the same intensity as the *S. aureus* SpsB positive control, indicating that the Nuc2 catalytic domain is present on the outside surface of the cell. Taken together, our findings demonstrate that *S. aureus* Nuc2 is localized to the cytoplasmic membrane and the C-terminal enzyme domain faces the extracellular environment, matching computational predictions.

### Nuc2 activity can be measured at the cell surface

**Figure 3:**
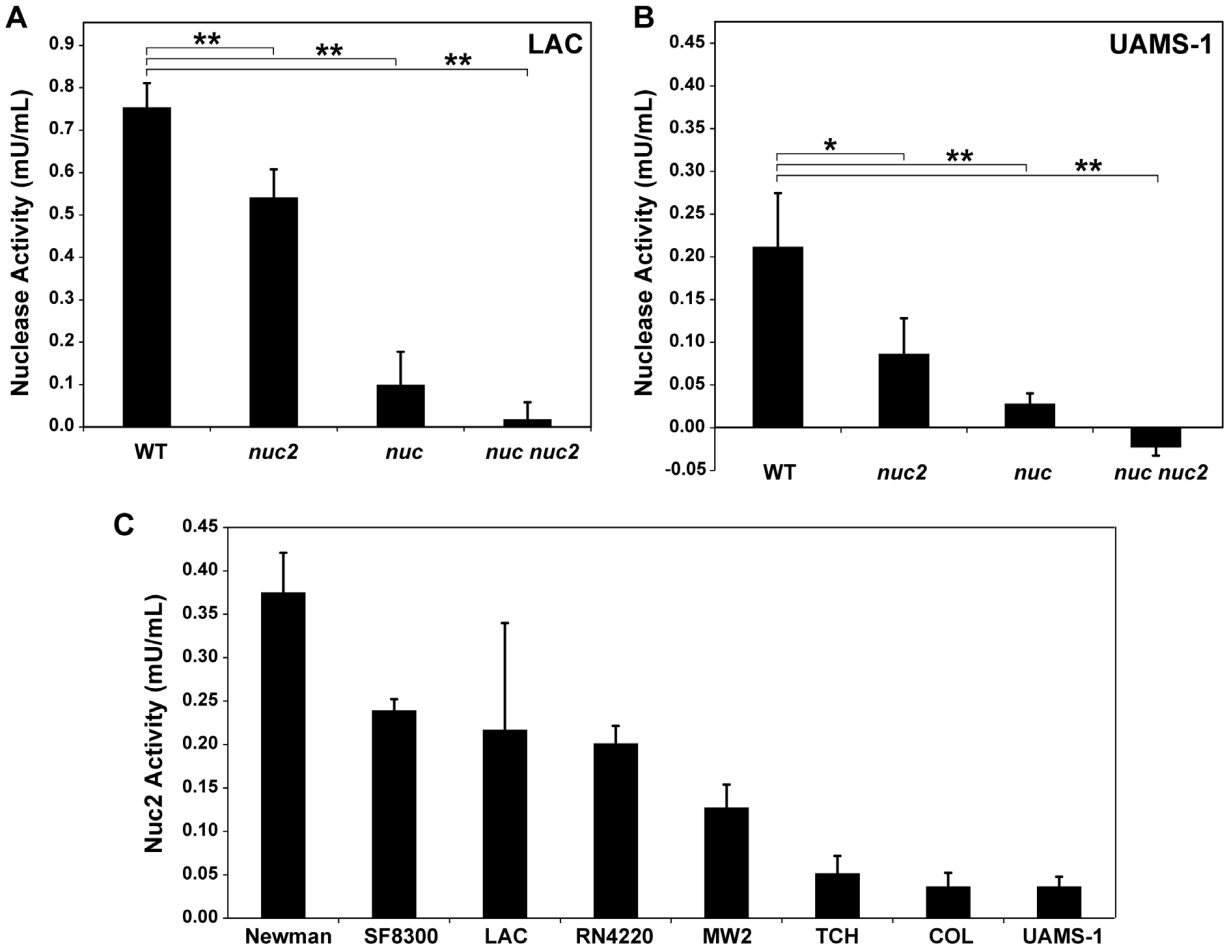
Nuc2 activity can be measured at the cell surface. **A**. Whole-cell FRET activity assay measuring Nuc2 surface function with UAMS-1 WT, *nuc2*, *nuc* and *nuc nuc2* double mutants. **B**. Cell surface nuclease activity in USA300 strain LAC WT, *nuc2*, *nuc* and *nuc nuc2* double mutants. Statistical significance (*p<0.1; **p<0.01) was determined by a Student's T-test. **C**. A collection of *S. aureus nuc* mutants tested in the same whole-cell FRET assay. The strains represented several genetic backgrounds, including (from left) Newman, USA300 strains (SF8300 and LAC), RN4220, MW2, TCH1516, COL and UAMS-1.

Mutation of the *nuc* gene in several strain backgrounds abolishes nearly all observable extracellular nuclease activity when culture supernatants or cell cultures were spotted onto methyl green DNase agar plates. However, faint halos were observed with low reproducibility around culture spots of some *S. aureus nuc* mutants (data not shown), suggesting the presence of nuclease activity in the *nuc* mutants that might be attributed to Nuc2. We hypothesized that one explanation for the lack of reproducibility could be that Nuc2 activity levels are near the nuclease detection limit of methyl green agar plates. In order to address this possibility, we adapted a sensitive fluorescence resonance energy transfer (FRET) assay previously used to detect Nuc activity as low as 0.015 U/ml in conditioned media [18], with a goal of measuring nuclease activity on the surface of whole cells. Two sets of four *S. aureus* strains in the UAMS-1 and LAC (AH1263) background were assembled that included wild-type (WT), *nuc* and *nuc2* single mutants, and *nuc nuc2* double mutants. These eight strains were compared using the modified whole-cell FRET assay. Low levels of nuclease activity could be detected on the surface of both *S. aureus nuc* mutants (∼0.1 mU/mL), which is an approximately 4.5-11 million fold reduction in activity compared to spent media from WT strains (assuming the reported levels of 452 U/mL for UAMS-1 and 1100 U/mL for LAC [18]). In each case, this activity was eliminated in a *nuc nuc2* double mutant, demonstrating Nuc2 activity is measurable using the whole-cell assay. We note that residual Nuc activity was also observed on the cell surface in this assay (Fig. 3A, 3B). This is presumably due to either incomplete washing or the slow release of Nuc from cells. Thus using the whole-cell assay method, Nuc2 extracellular nuclease activity was detected on the *S. aureus* surface for the first time.

We next examined different *S. aureus* strains to determine whether Nuc2 activity varies across strain backgrounds. A collection of *S. aureus nuc* mutant strains was tested in the whole cell FRET assay (Fig. 3C). *S. aureus* Newman exhibited the highest level of surface-associated extracellular nuclease activity (>0.3 mU/mL), followed by the USA300 strains SF8300, and LAC, and strain RN4220, which had ∼0.2 mU/mL of nuclease activity. Strains MW2 (USA400), TCH1516 (USA300), COL and UAMS-1 all possessed low levels of surface activity <0.15 mU/mL.

### Purified Nuc2 has nuclease activity and similar properties to Nuc

**Figure 4:**
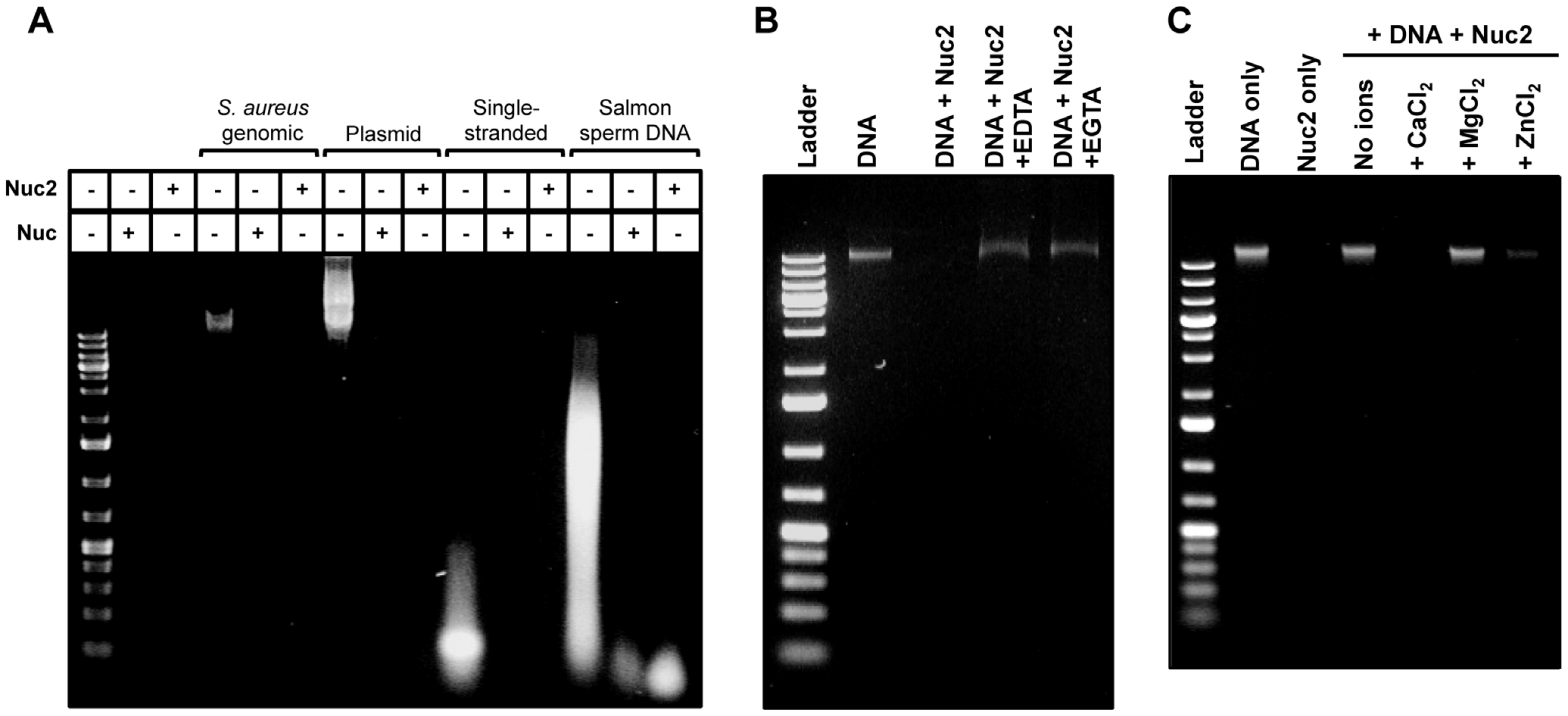
Purified Nuc2 has nuclease activity. Agarose gel degradation assays were performed on a number of different conditions. **A**. Activity of purified Nuc2 and Nuc against a variety of DNA substrates, including *S. aureus* and *E. coli* genomic DNA, single-stranded DNA, plasmid DNA, and eukaryotic DNA. **B**. Activity of Nuc2 against genomic DNA in the presence of chelators EGTA and EDTA. **C**. Nuc2 protein was stripped of metal ions and reconstituted with either 10 mM CaCl_2_, MgCl_2_ or ZnCl_2_ buffers.

To better characterize Nuc2 activity, Nuc2 enzyme lacking the N-terminal membrane anchor was purified using nickel-affinity chromatography. The activity of purified, his-tagged Nuc2 was determined by both a FRET-based assay and agarose gel DNA degradation assays. Nuc2 effectively cleaved the single-stranded DNA substrate in the FRET assay in a dose-dependent manner (data not shown). Agarose gel assays showed that Nuc2 was capable of degrading several nucleotide substrates, including *S. aureus* genomic DNA, eukaryotic DNA (salmon sperm), double-stranded plasmid DNA, and single-stranded DNA (Fig. 4A).

The cation-dependence and thermostability of Nuc2 were also assessed. Since Nuc requires the binding of a divalent calcium ion for activity [12], we hypothesized that Nuc2 would also have similar metal requirements. The chelators EGTA and EDTA prevented Nuc2 degradation of *S. aureus* genomic DNA, indicating that Nuc2 activity is cation-dependent (Fig. 4B). To determine the specificity of divalent cation, the Nuc2 protein preparation was stripped of metal ions using chelating resin (see Methods), and the cation-free protein was reconstituted with buffer containing either CaCl_2_, MgCl_2_ or ZnCl_2_. Complete degradation of bacterial genomic DNA with reconstituted Nuc2 was observed only with the addition of CaCl_2_ to the buffer, thus demonstrating that active Nuc2 requires calcium (Fig. 4C), while partial degradation of DNA was achieved with the addition of ZnCl_2_. To determine if Nuc2 is also thermostable, samples of Nuc and Nuc2 enzyme were heat-treated at 75°C for 1 hr and tested for the ability to degrade bacterial genomic DNA. Both heat-treated Nuc and Nuc2 were able to degrade the DNA substrate, while heat-treated Bovine DNaseI was not (data not shown). Taken together, the substrate preferences of Nuc2, calcium requirement, and thermostability, all mirror those of Nuc. The experimental findings that Nuc2 and Nuc are similarly thermostable differ from the recent observations by Hu and colleagues [20].

### Nuc2 is expressed at low levels during S. aureus growth

**Figure 5:**
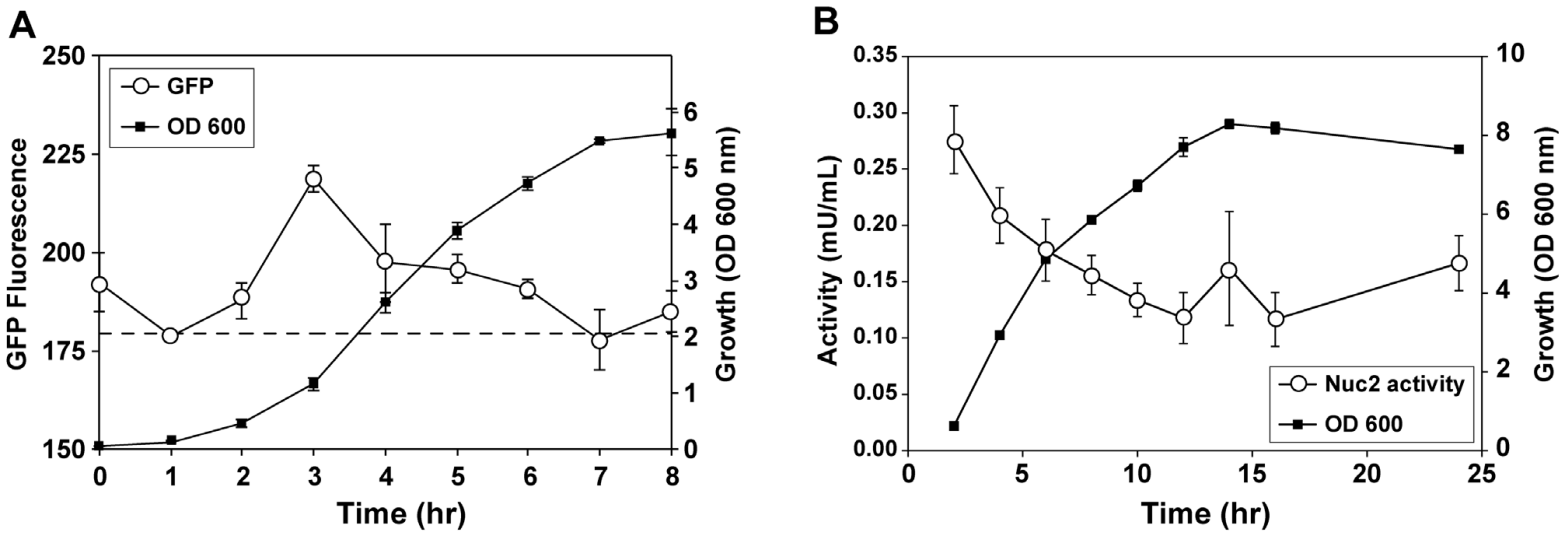
Nuc2 is expressed at low levels during growth. **A**. Monitoring changes in fluorescence of a P*_nuc2_*-sGFP transcriptional reporter over time (open circles) in comparison to growth (black squares). Dotted line indicates level of background fluorescence of growth media. **B**. Changes in surface Nuc2 activity (open circles) present in a *S. aureus* USA300 LAC *nuc* mutant over time as measured by whole-cell FRET assay.

Previous reports indicated that *nuc2* expression is highest during early log-phase growth and regulated by different mechanisms than *nuc* [21]. We assessed *nuc2* expression by fusing the native promoter region to the sGFP coding sequence, and the expression from this P*_nuc2_*-sGFP reporter in *S. aureus* was measured in a time course with cells grown in TSB at 37°C aerobically (Fig. 5A). A low level of fluorescence was observed that peaked slightly after the first 3 hr of logarithmic growth and declined somewhat as the cells approach stationary phase. Multiple P*_nuc2_* fusion constructs with upstream promoter regions of varying length were tested, and all behaved in a similar manner (data not shown). In an attempt to uncover a growth condition that modulates *nuc2* expression, the P*_nuc_*2-sGFP reporter strain was grown under varying nutrient, temperature, and oxygenation conditions, all without a change in the reporter kinetics. Conditions tested include growth at various temperatures (30°C, 37°C and 42°C), various media (TSB, brain-heart infusion (BHI), Todd Hewitt, LB, B2, Terrific, and Mueller Hinton broth, as well as BHI or TSB supplemented with 0.2% and 0.4% glucose), and static versus shaking conditions. To assess the impact of global regulators, the *nuc2* reporter plasmid was transformed into a collection of 15 *S. aureus* two-component system mutants [38], and *sarA* and *sigB* regulatory mutants. SarA and SigB are known to repress *nuc* expression [18], while SaeRS is required for *nuc* expression [16]. Again, the expression profile of the *nuc2* reporter was unchanged in the TCS and regulatory mutants when grown in TSB at 37°C (data not shown).

To determine if enzyme activity correlates with *nuc2* expression, a time course experiment was performed using the whole-cell FRET assay to detect cell-surface nuclease activity. The experiment was performed using a *S. aureus nuc* mutant to eliminate the overlapping contribution of Nuc to the assay. Similar to the *nuc2* transcription time course (Fig. 5A), Nuc2 activity on the surface is highest during early logarithmic growth (Fig. 5B) and drops as the cells enter stationary phase.

### Nuc2 is expressed in vivo

**Figure 6:**
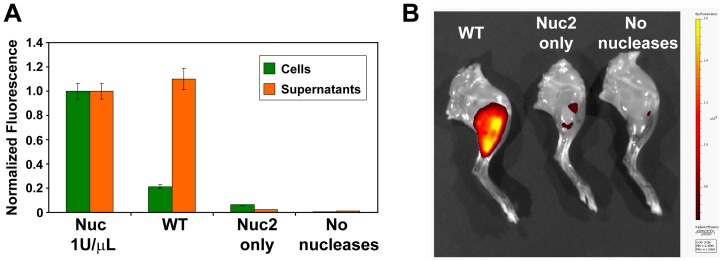
Nuc2 activity is detectable in mouse infections. **A**. Activation of the TT probe by recombinant micrococcal nuclease (left) or spent culture media (orange) or cell suspensions (green) of *S. aureus* WT, a strain only expressing Nuc2 (*nuc* mutant), and strain without nucleases (*nuc nuc2* double mutant). **B**. TT probe activation at infection sites of leg muscle tissue of mice bearing infections with the same three strains. This image is representative of 3 independent experiments.

We recently developed a new method to detect *S. aureus* nuclease activity *in vivo* using an activatable probe [19]. This entails intravenous administration of a quenched fluorescent oligonucleotide probe (i.e., a FRET probe) in a mouse model of pyomyositis that is susceptible to digestion by Nuc, but resistant to serum nuclease digestion, followed by fluorescence imaging. The probe utilized for these studies has a central pair of deoxythymidine residues (and is thus named “TT probe”) flanked with several 2′-*O*-methyl modified nucleotides. Two different TT probes were synthesized, one that includes a 5′-FAM fluorophore for *in vitro* assays, and a second that includes a 5′-Cy5.5 fluorophore that emits in the near-infrared portion of the spectrum for *in vivo* imaging. We repeated the whole cell assays with the TT probe and observed that most of the nuclease activity is lost in a *nuc* mutant (Fig. 6A), but some residual activity remained. By introducing a *nuc2* mutation, the remaining activity was eliminated, indicating that Nuc2 is functional and capable of digesting the TT probe, similar as observed with the DNA probes (Fig. 3).

Next, we sought to determine whether Nuc2 activity could be detected with the TT probe in the context of infections in animals. For this, we administered the probe to mice bearing WT, *nuc* or *nuc nuc2* double mutant in thigh muscle infections. After allowing 45 minutes for the probe to circulate and become activated, the mice were sacrificed, the legs were dissected to reveal the infections and examined with fluorescence imaging. As shown in Figure 6B, the probe was well-activated at the *S. aureus* WT infections. The residual activity seen in the *nuc* infections was further reduced in the double mutant, indicating that Nuc2 is responsible for the difference. Similar differences in probe activation were observed in images of animals taken prior to sacrifice (not shown). In summary, these data provide an indication that Nuc2 is active in the context of *S. aureus* infections *in vivo*.

### Activity of Nuc/Nuc2 chimeric proteins

**Figure 7:**
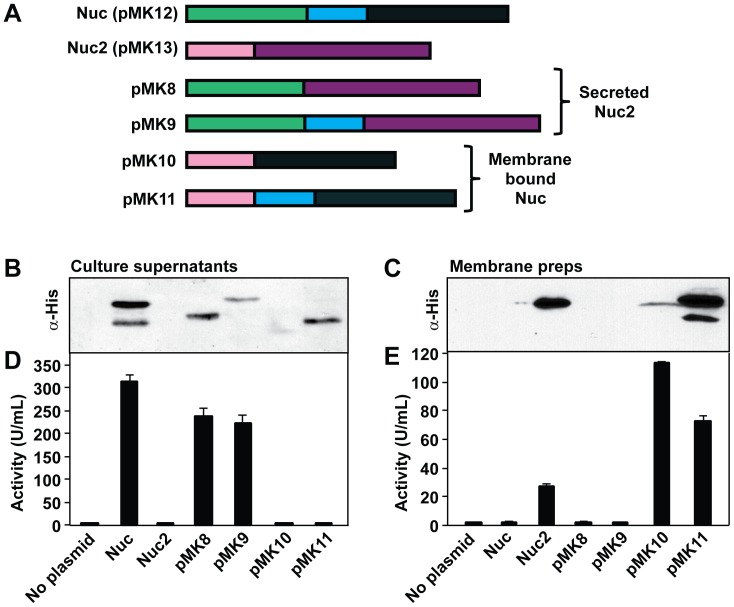
Activity of Nuc/Nuc2 chimeric proteins. Chimeras consisting of combinations of Nuc and Nuc2 N- and C-terminal protein domains were constructed. Each chimera was constructed to include a His_6_-tag on the C-terminal protein domain. **A**. Schematic showing the Nuc/Nuc2 chimeric proteins constructed, with Nuc domains colored in teal and Nuc2 domains colored in purple (see Fig. 1A for additional description of domains and processing sites). Chimeras constructed include, from the top down: Nuc control (pMK12), Nuc2 control (pMK13), Nuc signal sequence fused to the Nuc2 active domain (pMK8), Nuc signal sequence and NucB leader fused to the Nuc2 active domain (pMK9), Nuc2 membrane anchor fused to NucA (pMK10) and Nuc2 membrane anchor fused to NucB (pMK11). Each construct was expressed in a *S. aureus nuc* mutant (strain AH1680) for 15 hr, and culture supernatant was saved and membranes purified. Anti-His immunoblots were performed to detect the presence of C-terminal His_6_-tag on chimeric proteins in culture supernatants (**B**) or membrane (**C**) fractions. FRET activity assays were performed to detect nuclease activity in culture supernatants (**D**) and membrane (**E)** fractions. The *nuc* mutant strain was included as a negative control (called “no plasmid”).

Considering that Nuc and Nuc2 possess similar enzymatic activities, the major difference between the two enzymes appears to be the presence of a secretion signal sequence in Nuc versus the N-terminal membrane anchor in Nuc2 (Fig. 1). Due to the low Nuc2 activity measured at the *S. aureus* cell surface, we hypothesized that localization to the cell membrane could be impairing or masking Nuc2 activity. We constructed chimeric, tagged proteins to address this question. As part of the construct design, we also included the additional 19-residue segment that is only found in NucB (called “NucB leader”; Fig. 1A). Four chimeric His-tagged fusion proteins were constructed by splicing together: a) the N-terminal Nuc secretion signal or NucB leader to the Nuc2 C-terminal active domain, or b) the N-terminal Nuc2 membrane anchor to the NucA and NucB C-terminal active domains (see color-coding in Fig. 7A). The constructs were expressed under the control of a constitutive promoter in a *S. aureus nuc* mutant background, and nuclease activity in bacterial culture supernatants (Fig. 7D) and activity associated with purified cell membranes (Fig. 7E) was measured using the FRET assay. Anti-His immunoblots were performed on culture supernatants (Fig. 7B) and purified membranes (Fig. 7C) to confirm localization of fusion proteins.

When the Nuc2 C-terminal active domain was fused to the Nuc signal peptide, with or without the NucB leader, the fusion proteins were secreted (constructs pMK8 and pMK9; Fig. 7B), and activity was detected in the culture supernatant (Fig. 7D). Conversely, membrane anchoring by fusion of either NucA or NucB to the Nuc2 N-terminus did not impair nuclease activity, as high levels of nuclease activity could be measured in membrane preparations (constructs pMK10 and pMK11; Fig. 7E). Some aberrant behavior in the immunoblots was observed with the Nuc2-Nuc chimera (pMK10) being at a low level in the membrane (Fig. 7C), although nuclease activity was high (Fig. 7E). Similarly, a low amount of the Nuc2-NucB chimera (pMK11) was detected in culture supernatant (Fig. 7B), although nuclease activity was absent (Fig. 7D). Nonetheless, the presence of the Nuc2 leader and localization to the membrane does not notably alter nuclease activity of either Nuc or Nuc2 when constitutively expressed.

### Nuc2 is detrimental to S. aureus biofilms

**Figure 8:**
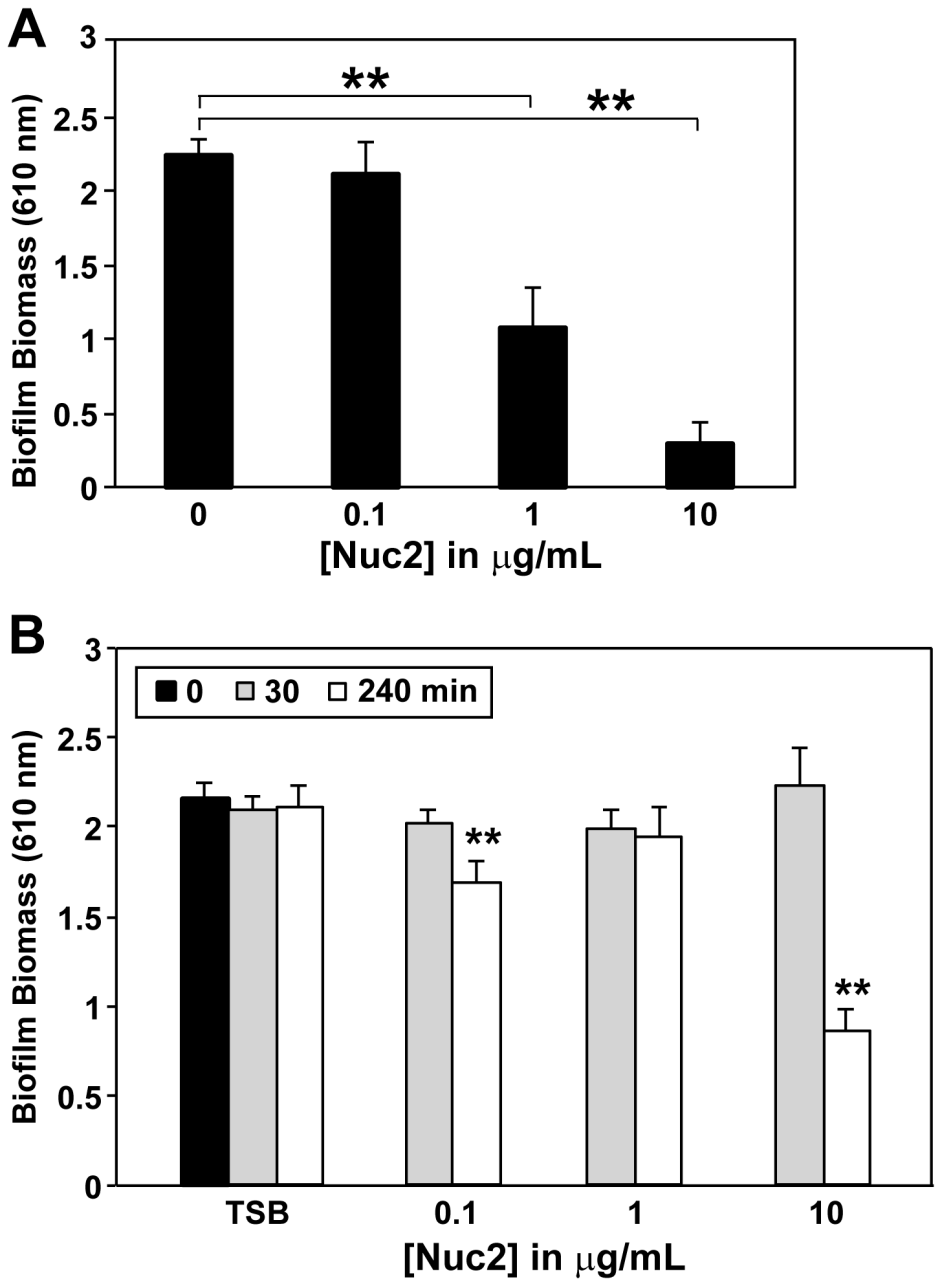
Effect of Nuc2 on *S. aureus* biofilms. **A**. Prevention of SH1001 biofilm formation in a microtiter plate assay by addition of purified Nuc2 at doses of 0, 0.1, 1, and 10 µg/mL at the time of inoculation. **B**. An established SH1001 biofilms is treated with Nuc2 and incubated for 0 (black), 30 (gray), or 240 (white) minutes. Results are representative of 3 trials, with 6 wells per strain measured per trial. Error bars show 1 standard deviation from the average absorbance measured. Statistical significance (**p<0.0001) was determined by a Student's T-test.

Another recently described property of Nuc is that it can prevent biofilm initiation [18]. We tested whether the exogenous addition of Nuc2 enzyme would have a similar impact on biofilm integrity. When *S. aureus* was incubated with purified Nuc2, the presence of Nuc2 could prevent biofilm formation in a dose-dependent manner (Fig. 8A). We also tested whether Nuc2 would have a negative impact on established *S. aureus* biofilms. The addition of Nuc2 to established biofilms had a mild dispersal effect, with higher amounts of Nuc2 able to disperse approximately 50% of the accumulated biomass after 4 hr of incubation (Fig. 8B). Thus, Nuc2 has anti-biofilm properties that mirror those of other DNases.

## Discussion

The discovery that *S. aureus* encodes a second thermonuclease has only recently been revealed [36]. Secreted Nuc is well studied, with much information available on its activity, chemistry and folding properties [10], [12]–[14], whereas the second nuclease, Nuc2, has received only limited attention. Nuc2 was initially evaluated in regulation studies, biofilm and enzyme assays [20]–[22], [36], and mutations in *nuc2* reduce the ability of *S. aureus* to form “giant colonies” on soft agar surfaces [39]. However, the high activity and secreted nature of Nuc masks the phenotypes of *nuc2* mutants, making it hard to investigate Nuc2 function in the extracellular environment. Thus, key questions about Nuc2 have remained, such as the localization of the enzyme in *S. aureus*, whether it is active in clinical isolates, and whether it is expressed *in vivo* during infection. With the emerging interest in using bacterial nucleases as imaging tools for monitoring infections [19], it is important to determine these basic properties of Nuc2 in order to assess this enzyme's contribution to the total extracellular nuclease capacity of *S. aureus*.

In comparing Nuc and Nuc2, the overall amino acid identity is high (34%), and in the catalytic domain the identity is even greater (42%). Structural predictions show striking similarities between the two proteins, with some minor differences in the disordered loops surrounding the active site (data not shown). However, Nuc2 is missing 2 of 9 active site residues previously shown to be required for Nuc activity, and the N-termini of the two proteins vary greatly, with Nuc possessing a secretion signal and Nuc2 a membrane anchor. With these preliminary observations, it was unclear as to whether Nuc2 would behave similarly to Nuc or have a completely different function in *S. aureus*. By modifying a previously developed FRET-based assay for nuclease activity [18], we demonstrated that Nuc2 is in fact an active enzyme on the surface of *S. aureus*, and Nuc2 activity is present in all tested strains. Localization studies confirmed the enzyme is membrane bound, with the active site facing the extracellular environment, indicating Nuc2 is a signal-anchored Type II membrane protein [35]. Using purified enzyme, we demonstrated that Nuc2 closely resembles Nuc with regard to substrate preferences, heat stability, and calcium dependence, which mostly supports a recent Nuc2 report [20].

We hypothesize that the correct conditions have not yet been identified to reveal a robust phenotype for a *nuc2* mutant, and our efforts to investigate gene regulation support this statement. The *nuc2* gene is transcribed early in log-phase growth, peaking approximately 3 hours post-inoculation, and this parallels enzyme activity measurements (Fig. 5). Studies of Nuc-Nuc2 chimeric fusion proteins begin to address the question of low Nuc2 activity in *S. aureus* by providing preliminary regulatory information. When *nuc2* was placed under the control of a constitutive promoter (plasmid pMK13), high levels of surface nuclease activity were measured (Fig. 7E). Taken together with the result that membrane localization does not impair Nuc activity, these observations suggest that the low Nuc2 activity in WT strains might be a regulatory phenomenon. The promoter regions preceding *nuc* and *nuc2* were compared (data not shown), and the *nuc2* promoter region does not contain known binding sites for either SarA [40] or SaeR [41], two known *nuc* regulators, suggesting that *nuc2* expression is regulated by different mechanisms than *nuc*. The use of a *nuc2* transcriptional reporter did not reveal any major regulators of *nuc2* expression, even after testing reporter activity in mutants of many well-characterized regulators and under varied growth conditions. Thus, there remain many unanswered questions about *nuc2* regulation in *S. aureus*.

We also examined Nuc2 activity in a mouse model of *S. aureus* pyomyositis. When the masking effect of Nuc is removed, Nuc2 enzymatic activity is detectable (Fig. 6), and this observation confirms the previous detection of background nuclease activity in a *S. aureus nuc* mutant using activatable probes [19]. Therefore, although *nuc2* expression is low, there is clearly active enzyme being produced on the surface of cells that may contribute to *S. aureus* growth and pathogenesis *in vivo*. While the infection experiments carried out here did not reveal obvious growth differences of the *nuc* vs. *nuc nuc2* double mutants, it seems plausible that these could become apparent in alternative infection scenarios.

Our regulatory and localization observations raise questions about the role of Nuc2 in *S. aureus*. The best-known membrane-localized DNases in bacteria are associated with natural competence systems, and these include *Streptococcus* spp. EndA and the *Bacillus subtilis* competence nuclease NucA [42], [43]. However, bioinformatics analyses show Nuc2 does not have homology to EndA or NucA, even though there is a recent report of competence in *S. aureus* [44]. It is also possible that a membrane-localized nuclease could serve a nutrient-scavenging role by degrading DNA to use as a carbon or phosphate source, as in *Shewanella oneidensis*, which encodes multiple extracellular DNases [45]. The potential role of one or both extracellular nucleases in nutrient acquisition warrants further study in *S. aureus*.

We have observed a detrimental effect of Nuc2 towards *S. aureus* biofilms when purified protein was added exogenously. It is possible that specific environmental conditions in host niches will modulate Nuc2 activity to facilitate dispersal from a biofilm. In previous studies, a slight up-regulation of *nuc2* was observed in *S. aureus* biofilms as compared to exponential- and stationary-phase cells grown in liquid culture [46]. In high-flow conditions, such as in infective endocarditis, there could be a benefit to having a surface-attached nuclease like Nuc2 due to the likelihood that secreted Nuc is cleared. Another possibility is that Nuc2 could digest eDNA in the biofilm matrix to allow for the formation of channels for the transport of nutrients and water into the biofilm and to allow waste products to be transported out of the biofilm [47], [48]. The inability of Nuc2 to achieve complete dispersal of pre-formed biofilms supports previous observations on the interactions of DNases with established biofilms. Dispersal of both *S. aureus* and *Pseudomonas aeruginosa* biofilms is time-dependent, with younger biofilms being DNase-susceptible while mature biofilms are more resistant [17], [49]. This phenomenon is thought to be a result of the increased amounts of proteins and polysaccharides present in an established biofilm matrix, coating and protecting the eDNA, while “younger biofilms” have more exposed eDNA that can be targeted by DNases.

Extracellular DNases in bacteria contribute in a variety of important ways to physiology and pathogenesis. In addition to the properties outlined above, bacterial DNases are critical for innate immune defense by degradation of NETs [15], [50], [51], a function that *S. aureus* Nuc2 might perform under certain *in vivo* conditions. At present, the exact contribution of Nuc2 to the *S. aureus* lifestyle as a commensal or pathogen remains unclear and will require further investigation. The results of this report will aid ongoing efforts to elucidate the function of Nuc2 in *S. aureus*.

## Supporting Information

Table S1 **Oligonucleotides used in this work.**
(DOC)
