# Supplementary material for: Staphylococcus aureus Nuc2 Is a Functional, Surface-Attached Extracellular Nuclease
Source: PLoS One. 2014 Apr 21;9(4):e95574. doi: 10.1371/journal.pone.0095574 (PMC3994088; doi:10.1371/journal.pone.0095574)
Supplement: Table S1 — Oligonucleotides used in this work. (DOC) [file pone.0095574.s001.doc]

**Table S1.** Oligonucleotides used in this work.

| **Primer** | **Sequence** | **Description** |
| --- | --- | --- |
| CLM342 | 5’-GTTGTTAAGCTTAACAATCAAGTAAACGATAAAGC- 3’ | *nuc2* promoter f |
| CLM348 | 5’- GCTGCCGCGCGGCACTAGTTTACTCCAAATATTTAATTTCTG-3’ | *nuc2* promoter r |
| CLM344 | 5’- CTAGTGCCGCGCGGCAGCATGAGCAAAGGAGAAGAACTTTTC-3 | sGFP f |
| CLM332 | :5’- GTTGTTGAATTCTTAGTGGTGGTGGTG-3’ | sGFP r |
| MRK7 | 5’ TGTTGAGCTC  AAAGGAACCCATATGAAGTCAAATAAATCGCTTGCTATGATTGTG 3’ | *nuc2* forward |
| MRK8 | 5’GGTGGTTCTAGATTTACTCCAAATATTTAATTTCTGTTGTTTAGC 3’ | *nuc2* reverse |
| MRK9 | 5’ TGTTGAGCTC  AAAGGAACCCATATGAAAAAAGAAATATTGGAATGGATTATTTCA 3’ | *spsB* forward |
| MRK10 | 5’ GGTGGTTCTAGATTTAGTATTTTCAGGATTGAAATTATGTTTAAATTC 3’ | *spsB* reverse |
| MRK38 | 5’ GTTGTTGGTACCCCTTTTTGAAAGGACCCGTATGAT 3’ | *P_nuc2_* reverse |
| MRK39 | 5’ GTTGTTAAGCTTGTAAACGATAA AGCCTACAAAG 3’ | *P_nuc2_* forward |
| MRK44 | 5’ Gttgttggatccaatcatacgggtcctttc 3’ | Nuc2 – forward |
| MRK45 | 5’ GTTGTTctcgagTTTACTCCAAATATTTAATTTCTGTTG 3’ | Nuc2 – reverse |
| MK52 | 5’- GTT ggt acc TAG GCA GGT TTT AAA C Atg aca gaa tac tta tta agt gct -3’ | Nuc for, SarA RBS |
| MK53 | 5’- CGT ATG ATT TGC ATT TGC TGA GCT ACT TAG ACT -3’ | Nuc S.S. rev |
| MK54 | 5’- CGT ATG ATT ACT ATA TAC TGT TGG ATC TTC AGA -3’ | NucB rev |
| MK55 | 5’- GCA AAT GCA aat cat acg ggt cct ttc -3’ | Nuc2 for, Nuc S.S. OL |
| MK56 | 5’- GTA TAT AGT aat cat acg ggt cct ttc -3’ | Nuc2 for, NucB OL |
| MK59 | 5’- GTT GGT ACC TAG GCA GGT TTT AAA C ATG AAG TCA AAT AAA TCG CTT GCT -3’ | Nuc2 TM for, SarA RBS |
| MK61 | 5’- Ttt caa ttt GCA ACT TCA ACT AAA AAA TTA CAT -3’ | NucA for, Nuc2 OL |
| MK62 | 5’- TGA AGT TGC AAA TTG AAA TGC TAA TAC ACC TAC -3’ | Nuc2 TM rev, NucA OL |
| MK63 | 5’- Ttt caa ttt TCA CAA ACA GAT AAT GGC GTA -3’ | NucB for, Nuc2 TM OL |
| MK64 | 5’- TGT TTG TGA AAA TTG AAA TGC TAA TAC ACC TAC -3’ | Nuc2 TM rev, NucB OL |
| MK65 | 5’- gtt gtt gaa ttc tta gtg gtg gtg gtg gtg gtg TTA TTT ACT CCA AAT ATT TAA TTT CTG -3’ | Nuc2 rev, 6xHis |
| MK66 | 5’- gtt gtt gaa ttc tta gtg gtg gtg gtg gtg gtg TTG ACC TGA ATC AGC GTT -3’ | Nuc rev, 6xHis |
